# Assessment of cytotoxicity of some synthetic compounds against breast carcinoma spheroids with subsequent analysis of pro-apoptotic and gene expression

**DOI:** 10.1038/s41598-025-26942-w

**Published:** 2025-11-26

**Authors:** Khaled Mahmoud, Elham M. Youssef, Farid M. Sroor, Walid Fayad

**Affiliations:** 1 Drug Bioassay-Cell Culture Laboratory, Pharmacognosy Department, Pharmaceutical and Drug Industries Division, National Research Centre, Dokki, Giza, 12622 Egypt; 2 Biochemistry Department, National Research Centre, Giza, Egypt; 3 Organometallic and Organometalloid Chemistry Department, National Research Centre, Cairo, 12622 Egypt

**Keywords:** Synthetic compounds, Breast cancer, 2D, 3D spheroids model, Inflammatory, Apoptotic genes, Biochemistry, Biotechnology, Cancer, Drug discovery

## Abstract

The current study examined the anticancer potential of 80 synthetic organic compounds at 50µM using in vitro two-dimensional (2D) and three-dimensional (3D) spheroids model breast adenocarcinoma (MCF7) models. The most promising six cytotoxic compounds on the 3D spheroids model (> 70% growth cytotoxicity) were counter-screened for their safety on normal cells (RPE1 cell line) grown as 3D spheroids. In parallel, the six compounds were tested for their pro-apoptotic activity using the M30 Cyto death ELISA assay. Based on the activity on MCF7 3D spheroids model, half maximum inhibitory concentration (IC_50_ < 26µM), the safety on normal cells (< 35% cytotoxicity) and the pro-apoptotic activity on cancer cells (> fourfold increase of negative control), three compounds were considered the most promising and were selected for further investigation, named: F03, F06 and C02. These three candidates underwent further gene expression profiling of apoptosis and inflammatory related genes by quantitative real-time polymerase chain reaction (qRT-PCR). The gene expression results for the three compounds indicated favorable pro-apoptotic activity while unfavorable pro-inflammatory induction. Statistical analysis of the Xlog P values of the 3D spheroids model hits compared to that of the 80-compounds library showed a significant tendency for higher XlogP values in the 3D spheroids model hits sub-group, most probably due to the higher penetrability of lipophilic compounds into the tumor mass. The presented work can be considered a pilot model for an approach in anticancer drug discovery.

**Supplementary Information:**

The online version contains supplementary material available at 10.1038/s41598-025-26942-w.

## Introduction

Breast cancer is the most prevalent cancer among women worldwide^1,2^. It arises when normal cells acquire genetic and epigenetic alterations that enable uncontrolled growth and survival^3,4^. The major clinical challenge is metastasis, which significantly reduces survival rates^5–7^. Since there are substantial variations in the outcomes of early breast cancer among different regions, the burden of metastatic breast cancer (MBC) may differ from that of early-stage disease^8,9^.

Tumor development is regulated by the balance between cell proliferation and programmed cell death (apoptosis)^10–13^. Members of the Bcl-2 protein family play central roles in apoptosis regulation, acting either as pro-apoptotic or anti-apoptotic factors^14–16^. In parallel, inflammation strongly contributes to tumor progression, mediated by cytokines such as TNF-α, IL-6, and IL-8, along with matrix metalloproteinases like MMP-1 that remodel the extracellular matrix and promote cancer progression^17–19^.

Current breast cancer therapies include surgery, radiotherapy, and chemotherapy; however, their limitations highlight the need for safer and more effective agents^20,21^. High-throughput screening of synthetic compound libraries provides a promising strategy to identify molecules with selective cytotoxicity against cancer cells while sparing normal cells. Importantly, functional groups such as aromatic rings, sulfur moieties, hydrazine linkages, and thioamide groups may enhance compound–protein interactions, contributing to apoptosis induction and therapeutic potential^22–24^.

The present study aimed to evaluate the anti-cancer potential of 80 pre-plated synthetic organic compounds using both 2D and 3D spheroid models. The most active compounds were further assessed for their pro-apoptotic activity, safety on normal spheroids, and their effects on apoptosis-related genes (P53, BAX, BCL2) as well as inflammatory markers by RT-PCR.

## Results and discussion

### Cytotoxicity evaluation on MCF7 monolayers

The eighty compounds were screened at 50 µM. The results indicated that 30 compounds out of 80 proved cytotoxic, ranging from 70 to 100% with a ratio equivalent to 37.5% out of the total compounds. 16 compounds out of 80 proved cytotoxic, ranging from 40 to 69% with a ratio equivalent to 20% out of the total compounds. 34 compounds out of 80 proved cytotoxic less than 40% with a ratio equivalent to 42.5% out of the total compounds (Table 1). The results with negative signs indicate that the compound lacks efficacy and may exhibit carcinogenic activity.

**Table 1:** Mean % cytotoxicity of chemical compounds on the breast cancer monolayer model at 50µM.

Location in plate	Mean of % cytotoxicity ± SD	Location in plate	Mean of % cytotoxicity ± SD	Location in plate	Mean of % cytotoxicity ± SD
A02***	94.3 ± 0.06	A06***	82.4 ± 0.41	A010*	53.8 ± 4.9
B02***	100 ± 0.003	B06*	14.8 ± 2.6	B010*	0.0 ± 0.003
C02***	100 ± 0.003	C06*	0.0 ± 0.003	C010***	100 ± 0.003
D02*	5.2 ± 0.18	D06*	10.4 ± 0.6	D010*	52.1 ± 2.11
E02***	83.2 ± 1.2	E06*	26.7 ± 1.18	E010***	100 ± 0.003
F02***	100 ± 0.003	F06***	80.1 ± 1.7	F010***	100 ± 0.003
G02*	0.0 ± 0.003	G06***	93.4 ± 1.6	G010***	100 ± 0.003
A03***	94.2 ± 0.65	A07*	36.5 ± 1.2	A011**	44.2 ± 1.6
B03*	0.0 ± 0.003	B07*	0.0 ± 0.003	B011*	0.0 ± 0.003
C03***	86.6 ± 9.3	C07*	0.0 ± 0.003	C011***	83.1 ± 1.17
D03*	11.1 ± 0.9	D07***	88.7 ± 0.23	D011*	35.5 ± 0.35
E03***	80.3 ± 1.5	E07*	0.0 ± 0.003	E011***	93.7 ± 1.29
F03***	70.0 ± 4	F07*	4.4 ± 0.118	F011*	0.0 ± 0.003
G03**	49.8 ± 2.9	G07*	0.0 ± 0.003	G011*	15.6 ± 1.7
A04*	2.5 ± 0.35	A08*	17.2 ± 1	H02**	68.7 ± 2.7
B04*	-58.5 ± 0.003	B08*	8.5 ± 0.47	H03***	88.5 ± 5.8
C04*	14.5 ± 0.58	C08**	66.3 ± 4.3	H04**	46.0 ± 0.8
D04***	100 ± 0.003	D08***	97.7 ± 0.47	H05*	24.1 ± 1.17
E04**	62.6 ± 3.1	E08***	84.1 ± 1.6	H06*	7.8 ± 0.23
F04**	46.9 ± 2.6	F08***	100 ± 0.003	H07**	65.3 ± 2.3
G04*	0.0 ± 0.003	G08***	82.8 ± 0.76	H08*	18.0 ± 2.23
A05**	62.3 ± 2.5	A09***	100 ± 0.003	H09***	86.2 ± 1.7
B05***	91.3 ± 0.6	B09*	12.3 ± 0.7	H010*	27.5 ± 1.4
C05**	62.9 ± 3.8	C09*	9.9 ± 1.1	H011*	0.0 ± 0.003
D05**	49.7 ± 4.1	D09*	7.6 ± 0.2	doxorubicin	57.6 ± 2.1
E05**	47.5 ± 6.2	E09*	28.3 ± 0.9		
F05***	96.9 ± 1.4	F09*	9.9 ± 3.8		
G05**	65.7 ± 1.0	G09***	100 ± 0.005		

*Cytotoxicity less than 40%, **Cytotoxicity from 40 to 69%, ***Cytotoxicity from 70 to 100%, S.D Standard deviation.

### Cytotoxicity evaluation on multicellular spheroids of MCF7 and RPE1 cell lines.

As a relatively small library composed of 80 compounds was selected for this study, a relatively high concentration of 50 µM was applied for screening, to identify a reasonable number of hits, taking into consideration the resistance of the 3D spheroids model compared to the conventional monolayers. To compare the cytotoxic effect of compounds on monolayer versus 3D spheroids model, all compounds were screened on 3D spheroids model of human Caucasian breast adenocarcinoma cell line (MCF7). Results are presented in Table 2.

**Table 2:** Mean of % cytotoxicity of chemical compounds on 3D spheroids model MCF7 at 50µM.

Plate location	Mean of % cytotoxicity ± S.D	Plate location	Mean of % cytotoxicity ± S.D	Plate location	Mean of % cytotoxicity ± S.D
A02**	67.1 ± 3.3	D05**	50.9 ± 2.4	G08**	56.2 ± 3
B02*	35.7 ± 1.8	E05**	41.4 ± 3.6	H08**	49.0 ± 1.4
C02***	87.3 ± 4.5	F05*	31.6 ± 0.8	A09**	52.2 ± 1.9
D02	-8.0 ± 0.003	G05*	35.1 ± 0.6	B09**	59.2 ± 1.2
E02	-10.8 ± 0.7	H05*	19.3 ± 0.5	C09	-23.4 ± 0.5
F02**	52.0 ± 2.9	A06**	60.8 ± 1	D09**	50.0 ± 0.35
G02	-9.9 ± 0.8	B06*	20.4 ± 0.35	E09**	50.7 ± 0.52
H02*	3.5 ± 0.8	C06*	35.8 ± 0.23	F09*	14.2 ± 0.5
A03*	39.1 ± 0.24	D06**	40.8 ± 2.1	G09**	66.5 ± 1.1
B03*	25.5 ± 0.11	E06**	55.0 ± 1.6	H09***	80.8 ± 0.77
C03*	3.4 ± 0.17	F06***	88.0 ± 2.5	A010**	68.6 ± 1.1
D03*	23.5 ± 0.17	G06**	52.3 ± 2.3	B010**	48.2 ± 0.23
E03**	41.8 ± 0.6	H06*	26.1 ± 0.4	C010*	33.8 ± 1.6
F3***	91.5 ± 0.5	A07***	75.1 ± 0.53	D010**	51.7 ± 1.1
G03*	21.3 ± 0.6	B07*	34.9 ± 1.7	E010**	67.5 ± 0.5
H03*	15.8 ± 0.11	C07**	55.4 ± 2.3	F010**	60.3 ± 1.1
A04**	43.1 ± 0.23	D07**	50.1 ± 2.3	G010**	54.4 ± 1.2
B04**	49.3 ± 0.35	E07*	36.2 ± 0.35	H010**	60.6 ± 1.1
C04*	28.6 ± 0.76	F07**	54.2 ± 0.53	A011**	46.4 ± 1.3
D04**	67.9 ± 0.5	G07*	35.1 ± 0.77	B011*	30.5 ± 0.53
E04**	42.0 ± 1.1	H07**	40.6 ± 0.7	C011**	66.5 ± 0.24
F04*	29.8 ± 1.23	A08**	49.3 ± 0.53	D011**	40.6 ± 0.11
G04*	6.3 ± 0.3	B08**	53.1 ± 0.6	E011**	66.4 ± 1.9
H04*	28.0 ± 0.6	C08*	30.8 ± 1.3	F011**	60.4 ± 0.77
A05**	49.0 ± 0.8	D08***	74.0 ± 2.9	G011**	60.8 ± 1.23
B05	42.1 ± 1.9	E08**	40.2 ± 3.8	H011**	68.5 ± 0.65
C05**	48.6 ± 2.0	F08**	61.6 ± 0.8	cisplatin	70.1 ± 1.8

*Cytotoxicity less than 40%, **Cytotoxicity from 40 to 69%, ***Cytotoxicity from 70 to 100%

**Fig. 1:**
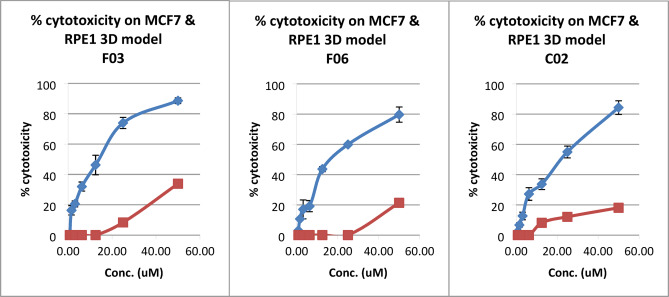
Dose response charts of the 3 most active compounds on MCF7 (blue) & RPE1 (red) 3D model.

Among the 80 compounds tested, 6 exhibited potent cytotoxicity (70–100%), accounting for 7.5% of the total. Another 45 compounds showed moderate cytotoxicity (40–69%), representing 56% of the compounds. The remaining 25 compounds demonstrated low cytotoxicity (< 40%), while 4 had negative signs as they increased the proliferation of the cells. To further assess their selectivity, the six most cytotoxic compounds were evaluated against a 3D spheroids model of normal RPE1 cells and their IC_50_ values were determined in a 3D spheroids model MCF7 cells. The percentage of cytotoxicity results in Table 3 indicates that compounds F03, F06, and C02 exhibit low cytotoxic effects on RPE1 spheroids at 50µM, with percentages ranging from 18% to 34.4%. These results encourage considering them for further investigation. On the other hand, compounds A07 and D08 showed significant cytotoxic effects on the normal spheroids, with percentages around 37.2% and 39.4%. Table 3 and Fig. 1 show varying levels of cytotoxicity among the tested compounds on RPE1 and their IC_50_ on MCF7 spheroids. Regarding our results, the three synthetic chemical compounds were considered as highly promising against human breast adenocarcinoma coded with F03, F06 and C02, with IC_50_ values of 18.8, 24.8, and 25.3 µM, respectively. The IC_50_ values of the six compounds can be confidently estimated as > 50 µM as all of them scored < 50% cytotoxicity at such a concentration. According to the literature, no data was reported for these three compounds as anticancer agents, up-till now.

**Table 3:** Mean of % cytotoxicity of promising compounds against 3D spheroids model RPE1 and IC_50_ of MCF-7 cell lines.

Compound code	Cytotoxic effect% ± SD RPE1(50µM)	IC_50_ (µM) for MCF7 ± SD
F03	34.4 ± 2.3	18.8 ± 1.3
F06	18.4 ± 0.5	24.8 ± 0.6
C02	18.3 ± 0.4	25.3 ± 1.2
A07	37.2 ± 1.2	31.7 ± 1.2
D08	39.4 ± 1	36.9 ± 0.6
H09	41.2 ± 1.6	41.2 ± 0.83
Cisplatin 40 µM	39.3 ± 3.0	31.7 ± 1.5

### Pro-apoptotic activity evaluation

**Fig. 2:**
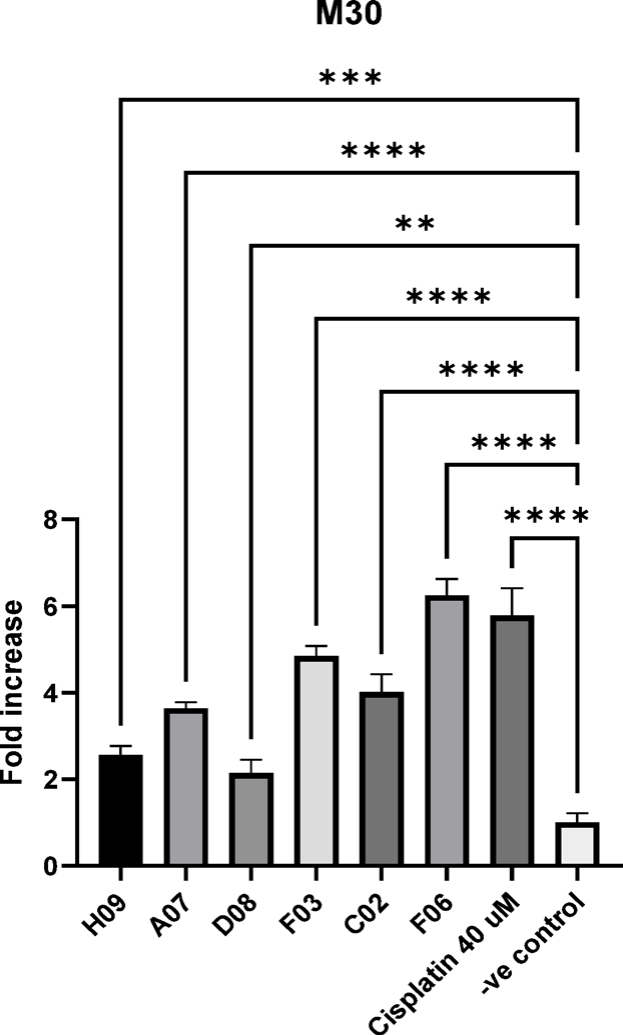
Pro-apoptotic activity of the six compounds measured by the M30 Cyto-death ELISA assay. The activity is represented as a fold increase of the negative control. Fold increase ≥ 4 is considered promising. Data are displayed as mean ± SD, n = 3. ** = *p* < 0.01, *** = *p* < 0.001, **** = *p* < 0.0001.

The results indicated that F06 induced sixfold increase, while F03 and C02 induced > fourfold increase in apoptosis signal compared to Dimethyl sulfoxide (DMSO) treated MCF7 cell line. These 3 compounds are considered the most active in apoptosis induction and were selected for further investigations. The pro-apoptotic activity is expressed in Table 4 and Fig. 2.

**Table 4:** Pro-apoptotic effect of promising compounds by using M30 cyto-death (ELISA assay) on MCF7 cell line.

Sample	OD 450	Fold increase	SD
H09	0.2311	2.56	0.21
A07	0.3289	3.64	0.14
D08	0.1933	2.14	0.32
F03*	0.439	4.85	0.23
C02*	0.3635	4.02	0.41
F06*	0.5655	6.25	0.38
Cisplatin (40 µM)	0.5231	5.78	0.64
DMSO	0.0904

The pro-apoptotic activity is measured as fold increase of the spontaneous apoptosis occurred in the DMSO treated negative control ± standard deviation.

The cutoff was determined as fourfold increase in the apoptosis signal of the negative control. The cutoff was chosen in alignment with the fold increase in apoptosis of 40 uM cisplatin, as positive.

control (4 times fold increase).

### Gene expression profile

**Fig. 3:**
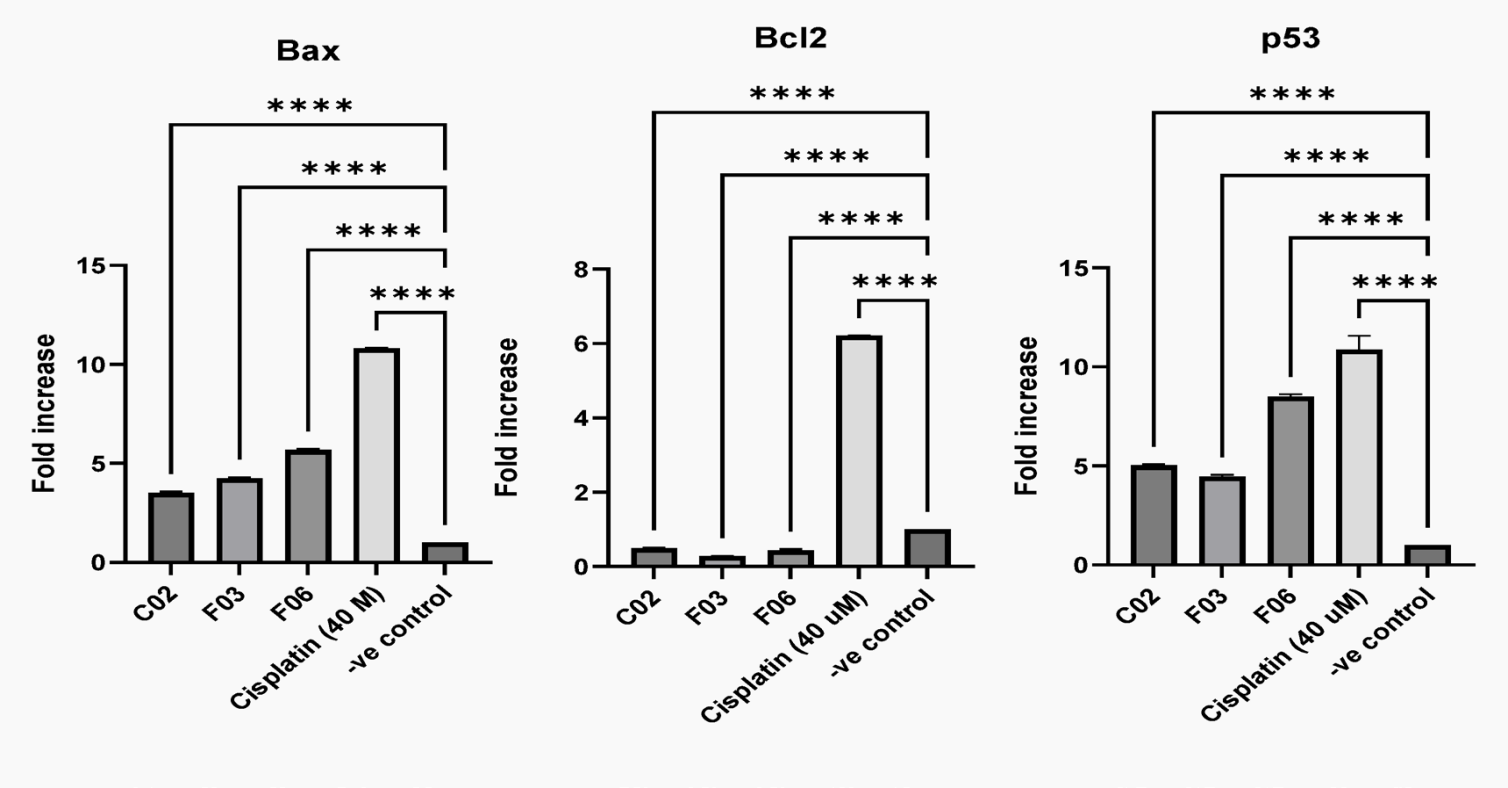
Apoptosis induction by three compounds through a mechanism involving the modulation of Bax, P53, and Bcl-2 expression. Data are displayed as mean ± SD, n = 3. **** = *p* < 0.0001.

Among the evaluated compounds, C02, F03, and F06 show the most potent pro-apoptotic activity. Of these, compound F06 emerges as a promising anti-cancer candidate due to its greater ability to downregulate Bcl-2 while upregulating both P53 and Bax expression compared to the other compounds. F03 and C02 exhibit a moderate pro-apoptotic effect compared to the positive control (Fig. 3). The three compounds, C02, F03, and F06, possess distinct bioactive moieties, including sulfonamide, urea, amide, thiourea, sulfur, thiophene, thiadiazole, and indole. Notably, the presence of sulfur, a redox-active element, is a common feature in these compounds.

**Fig. 4:**
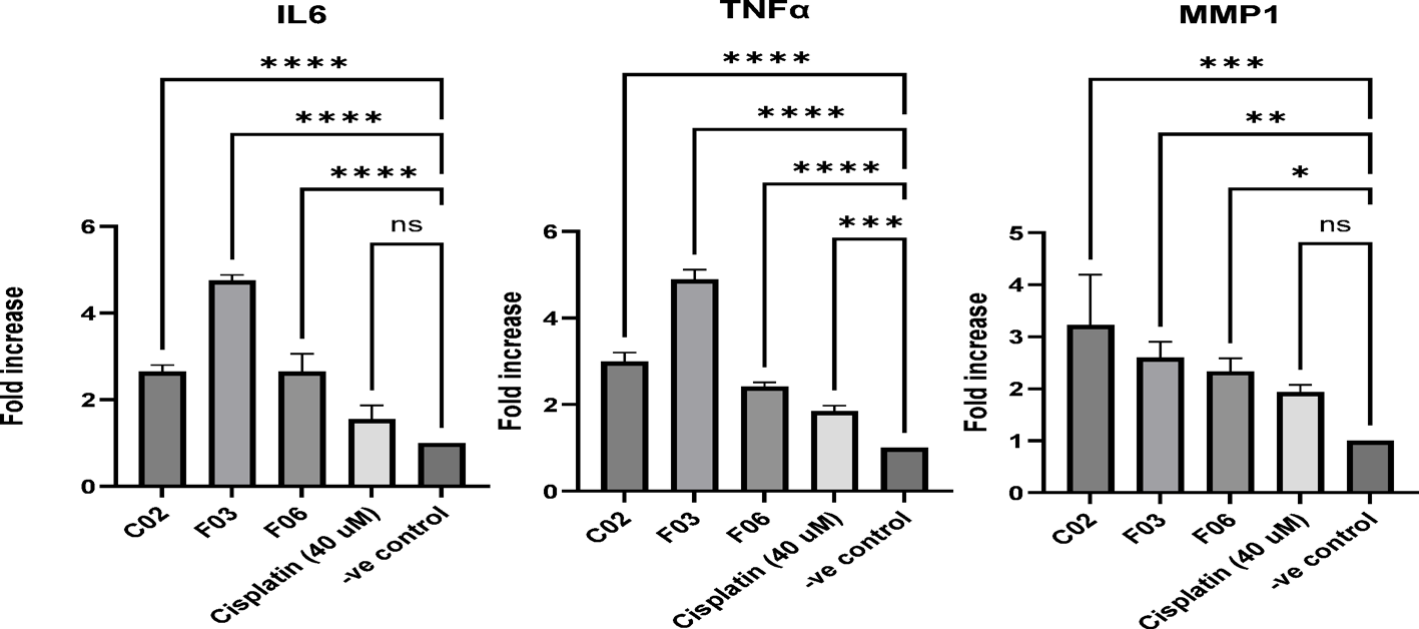
Effects of the three compounds on inflammatory markers in MCF7 Cells. Data are displayed as mean ± SD, n = 3. Ns = non-significant, * = *p* < 0.05, ** = *p* < 0.01, *** = *p* < 0.001, **** = *p* < 0.0001. IL-6: Interleukin 6; TNFα: Tumor necrosis factor alpha; MMP1: Matrix metalloproteinase-1.

The three compounds exhibited a moderately unfavorable pro-inflammatory effect, as indicated by their relatively increased expression of inflammatory markers IL-6, TNFα, and MMP1. In the context of a chemotherapy-oriented study, cisplatin was used as a positive control, as it is reported that it causes an inflammatory effect by triggering oxidative stress and activating pro-inflammatory signaling pathways, such as NF-κB, which increases the expression of inflammatory cytokines like IL-1β, IL-6, IL-8, and TNF-α. This results in tissue damage and the recruitment of immune cells, like neutrophils, leading to a wide range of cisplatin-induced toxicities in organs^25^. However, cisplatin showed milder pro-inflammatory effects compared to the tested three compounds. The gene expression results of the three compounds are expressed in Fig. 4.

### Determination of the XLogP values

**Fig. 5:**
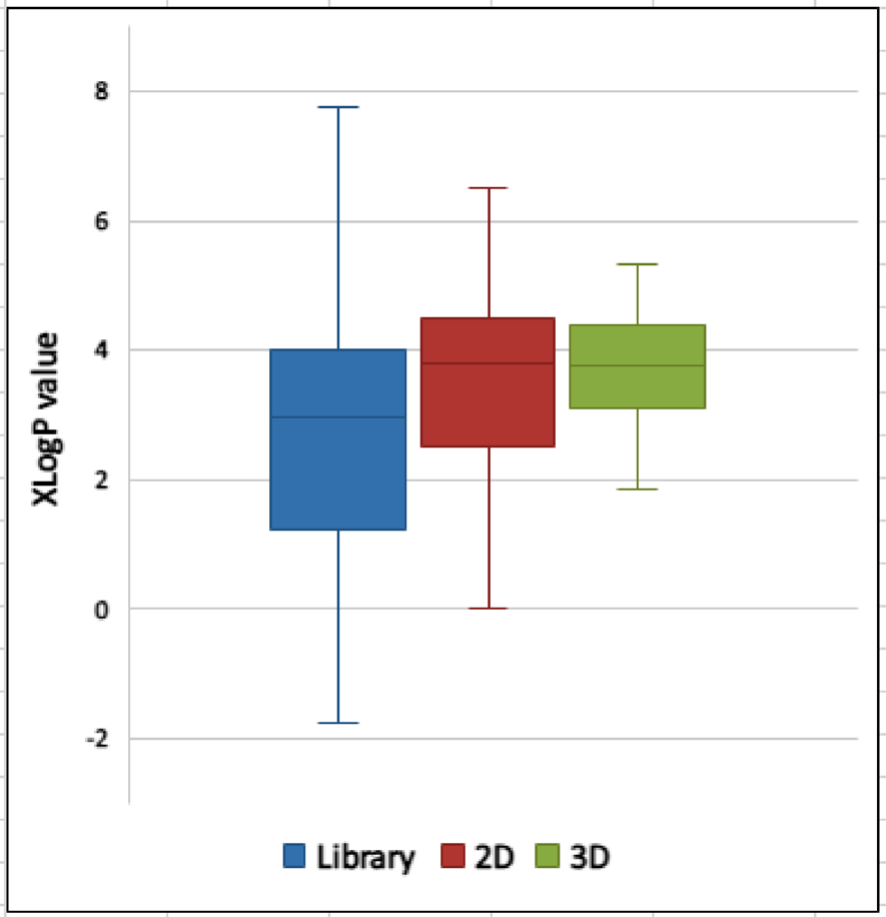
Distribution of XLogP values within the library (80 compounds), 2D and 3D hits.

In order to determine if there is a correlation between the lipophilicity of the compounds and the anticancer inhibitory activity, the XLogP values were calculated for the whole 80 compounds and for the active compounds on cancer monolayers and spheroids with > 70% cytotoxicity as separate two subsets (30 compounds in 2D and 6 in 3D). The ChemDraw software was utilized for XLogP values determination. The Mann–Whitney u test was applied to determine if there is a significant difference between the XLogP values for the hits compared to the 80 compounds. The results showed that there is a significance difference between the XLogP values for the 3D hits compared to the whole library at *p* < 0.055. A one tailed option was selected as the literature supports that lipophilicity is positively correlated with the penetration of the tumor mass and consequently anticancer activity. However, it is worth to clarifying that lipophilicity is not the only factor controlling the efficacy of an anticancer compound. The XlogP values of the spheroid hits is presented in Table 5, and their distribution within the library, 2D and 3D hits is illustrated in Fig. 5.

**Table 5:** The XLogP values of selected compounds (F03, F06, C02, D08, A07, and H09), are indicators of their potential effectiveness based on hydrophobicity and lipophilicity.

Compound	XLog*P* value
F03	4.53
F06	3.92
C02	3.61
D08	2.96
A07	5.32
H09	1.87

The data highlights 3 synthetic chemical compounds that appear to be particularly promising. These compounds demonstrated high cytotoxicity against the 3D tumor model, while showing relatively low toxicity to normal human cells. Importantly, these 3 compounds appear to be novel, with no previous reports of their anti-cancer activity according to previous literature.

**Fig. 6:**
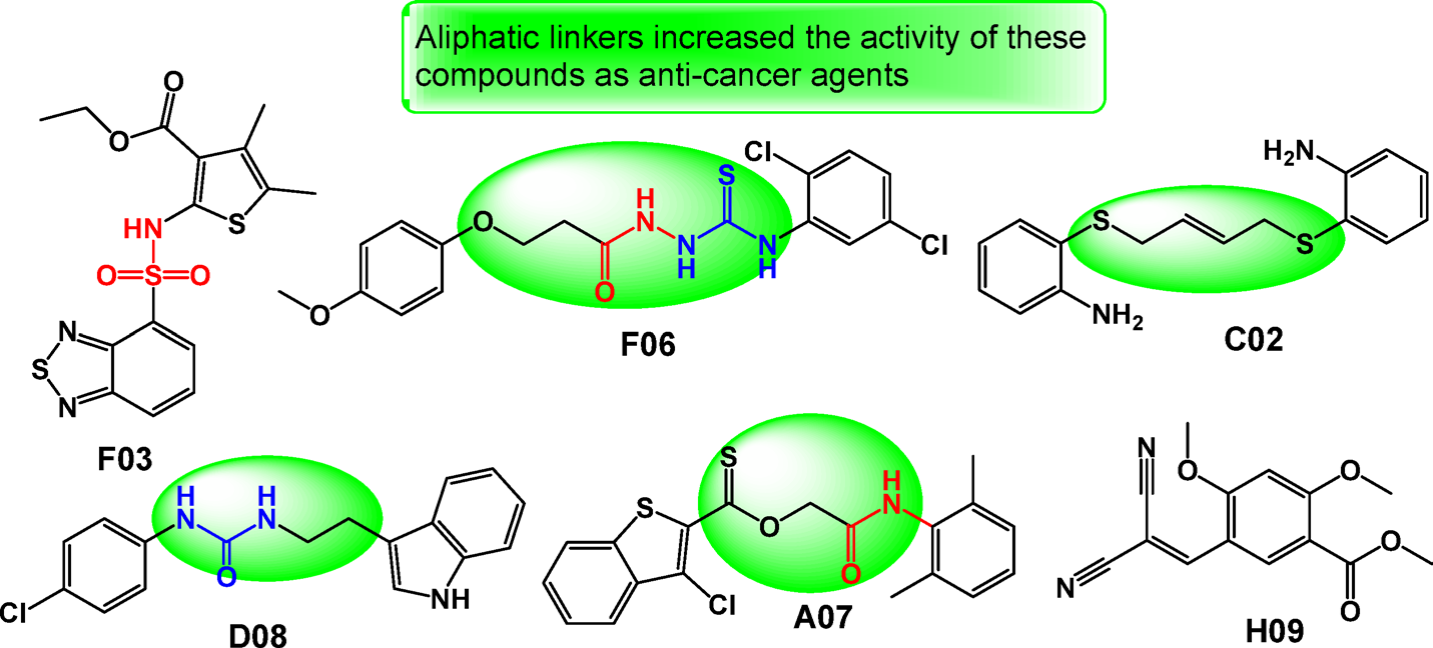
Chemical structures of compounds F03, F06, C02, D08, A07 and H09.

As depicted in Fig. 6, the chemical structure of all the most active compounds (F03, F6, C2, D8, A7, and H9) contains different bioactive moieties such as sulfonamide, urea, amide, thiourea, sulfur, thiophene, thiadiazole, and indole. The incorporation of aliphatic linkers and sulfur-containing moieties has been a well-established strategy in the discovery of biologically active organic compounds (F06, C02, D08 and A07)^21,26–29^. These activities of the promising compounds may be due to the presence of aliphatic linkers and sulfur-based moieties (Fig. 6).

Aliphatic linkers play a crucial role in modulating the physicochemical properties and enhancing the drug-like characteristics of bioactive compounds. These flexible, non-polar linkers can improve solubility, membrane permeability, and metabolic stability, all of which are essential for the effective delivery and absorption of potential drug candidates. Furthermore, the appropriate positioning and length of the aliphatic linker can facilitate favorable interactions with target biomolecules, thereby optimizing binding affinity and potency^30–34^. The XLogP values ranged from 1.87 to 5.32, which is an indication of effectiveness in biological system based on their structure^35^.

On the other hand, the presence of sulfur is another structural feature that has been extensively exploited in the design of biologically active compounds (F03, F06, C02 and A07). Sulfur-containing functional groups, like sulfonamide, thiourea, thiophene, and thiadiazole, can participate in various non-covalent interactions, such as hydrogen bonding, halogen bonding, and dipole–dipole interactions, which can enhance target binding and modulate the biological activity of the molecules^36,37^. Additionally, the redox-active nature of sulfur moieties allows them to play important roles in cellular signaling pathways and antioxidant mechanisms, contributing to the therapeutic potential of sulfur-containing drug candidates. The strategic incorporation of aliphatic linkers and sulfur-based substituents has been a common approach in medicinal chemistry, leading to the development of numerous clinically approved drugs and drug candidates across diverse therapeutic areas^38–41^. Additionally, the chemical structure collectively of the three compounds likely contribute to their anticancer properties by facilitating interactions with key biological targets involved in cancer progression. These features may enhance binding affinity, hydrogen bonding, and cellular uptake, ultimately leading to the inhibition of cancer cell growth and survival.

In summary, the cytotoxicity screening of the 80-compound library identified a broad spectrum of activities, ranging from highly potent to moderately and weakly active anti-breast cancer agents. Among the tested compounds: C02, F03 and F06 emerged as the most promising candidates, displaying superior cytotoxic activity compared to the other compounds. This finding suggests that it could serve as valuable lead molecules for the development of novel breast cancer treatment strategies. However, before its clinical relevance can be established, further investigations are essential to elucidate the molecular pathways and cellular targets underlying its anti-cancer effects, as well as evaluating its selectivity, safety profile, and efficacy in more advanced animal models.

## Materials & methods

Pre-plated organic compounds: This study explores the anti-cancer potential of 80 chemically diverse synthesized compounds prepared in Maybridge Thermofisher USA (Table S1). The compounds belong to the HitFinder screening collection with a purity greater than 90%^42^. The 80 compounds were designed and synthesized by the supplying company to have drug-like properties with chemical diversity. The compounds were tested according to the empirical approach and not the target-based approach. This project can be considered as a pilot study for larger screens where thousands of compounds can be tested, applying the same approach.

### Cell culture

Eighty pre-plated synthetic chemical compounds were screened on two human cell lines: MCF7 (ATCC® HTB-22™), a Caucasian breast adenocarcinoma model, and hTERT-RPE-1 (ATCC® CRL-4000™), a normal retinal epithelial cell line immortalized by hTERT telomerase. These cell lines were generously provided by Prof. Stig Linder, Karolinska Institute (Sweden). The cells were cultured in DMEM-F12 medium supplemented with L-glutamine and 10% fetal bovine serum, all were purchase from Biowest (Biotechnology company in Nuaillé, France), and maintained in a controlled incubator environment set at 37 °C with 5% CO2 and 95% humidity.

### Cytotoxicity evaluation on MCF7 monolayers

MCF7 cells were seeded in flat-bottom 96-well plates with a concentration of 20,000 cells/well, and were left overnight. The compounds were tested at a final concentration of 50 µM. Doxorubicin was used as a positive control and 0.5% DMSO as a negative control. The treatment duration was 72 h. The cytotoxicity was then assessed using the MTT assay, as described by Mosmann^43^.

### Cytotoxicity evaluation on multicellular spheroids of MCF7 and RPE1 cell lines

3-D spheroid models were generated from MCF7 cancer and hTERT-RPE1 normal cells were seeded at 10,000 and 50,000 cells/well, respectively, in poly-HEMA coated round bottom plates according to the protocol previously mentioned^44^. The cells were incubated for 5 days to generate the spheroids with diameter of 500 ± 20 µm. The spheroids were imaged using Olympus S100, U-CMAD3 camera connected to Olympus inverted microscope and the diameter of the spheroids was measured using Cellscen-Entry software. Test compounds were added in triplicate to a final concentration of 50 µM and were incubated for 120 h. A 40 μM final concentration of cisplatin was used as a positive control, and 0.5% DMSO was used as a negative control. At the end of incubation, cytotoxicity was determined using the acid phosphatase method^45^. After washing twice with 250 μL PBS buffer, 100 μL of 0.1 M sodium citrate, 0.1% Triton X-100, p-nitrophenylphosphate (2 mg/ml) (Pierce Biotechnology Inc., Rockford, IL) was added to each well and incubated for 1.5 h at 37 °C. After incubation, 10 μL of 1N NaOH stop solution was added to each well, and absorbance was read at 405 nm. Cytotoxicity was calculated according to the following equation:

$$\left[ {{1 } - \, \left( {{\text{av}}\left( {\text{X}} \right)} \right)/\left( {{\text{av}}\left( {{\text{NC}}} \right)} \right)} \right] \, *{ 1}00$$

where: Av: average, X: absorbance of sample, NC: absorbance of negative control.

### Pro-apoptotic activity evaluation

The accumulation of CK18-Asp396 in treated MCF7 cell cultures was quantified using the M-30 Cytodeath® ELISA kit according to the manufacturer’s instructions. Briefly, the MCF7 cell line monolayers were exposed to the chemical compounds at a concentration of 50 µM. Following 24 h of incubation, the culture medium was supplemented with 0.1% NP40. Subsequently, aliquots of 25 µL from each well, containing CK18 released into the medium from attached and floating cells and cell fragments, were subjected to the ELISA assay. Cleaved CK18 levels were determined by measuring the absorbance at 450 nm^46^.

### Gene expression profiling

The expression levels of the pro-inflammatory cytokine genes (IL-6 and TNF-alpha), the matrix metalloproteinase gene (MMP1), the tumor suppressor gene (p53), and the apoptosis-related genes (Bcl-2 and Bax) were evaluated in MCF7 breast cancer cells using real-time PCR after exposure to the IC_50_ concentration of the three selected compounds for 24 h.

Total RNA was extracted from both control and treated cells using the RNeasy kit. Up to one million cells were subjected to disruption in Buffer RLT, homogenization, and ethanol treatment to promote selective binding of RNA to the RNeasy membrane. High-purity RNA was subsequently eluted using RNase-free water. All binding, washing, and elution steps were performed using centrifugation in a micro-centrifuge. The sample was then applied to an RNeasy Mini spin column, enabling total RNA retention on the membrane while contaminants were efficiently eliminated. The RNA was then used as a template for real-time PCR to quantify the mRNA levels of genes. Two sets of primers were used—one for the IL-6, TNF-alpha, MMP1, p53, Bcl-2, and Bax genes and another for the endogenous control gene, β-actin (Table 6). The real-time PCR reaction was carried out with the following cycling conditions: 50 °C for 45 min (cDNA synthesis), followed by 40 cycles of 95 °C for 10 s and 60 °C for 60 s. A negative control was included to ensure primer specificity and rule out contamination. Finally, the statistical analysis of the expression of the genes was performed using one-way ANOVA followed by the Tukey–Kramer post-test.

**Table 6:** Oligonucleotide primers used for real-time RT-PCR analysis.

Genes	Forward primer	Reverse primer
IL6	5’- TACCACTTCACAAGTCGGAGGC-3’	5’- CTGCAAGTGCATCATCGTTGTTC-3’
TNFα	5’-GGTGCCTATGTCTCAGCCTCTT-3’	5’-GCCATAGAACTGATGAGAGGGAG-3’
MMP1	5’-AGGAAGGCGATATTGTGCTCTCC-3’	5’-TGGCTGGAAAGTGTGAGCAAGC-3’
p53	5’- CCCCTCCTGGCCCCTGTCATCTTC-3’	5’-GCAGCGCCTCACAACCTCCGTCAT-3’
Bcl-2	5’-CCTGTGGATGACTGAGTACC-3’	5’-GAGACAGCCAGGAGAATCA-3’
Bax	5’-GTTTCATCCAGGATCGAGCAG-3’	5’-CATCTTCTTCCAGATGGGTGA-3’

## Statistical analysis

GraphPad PRISM software was used in this work. In cytotoxicity on 2D and 3D models, non-linear regression analysis was applied. For pro-apoptotic and gene expression experiments, one way ANOVA was used followed by Dunnett’s Multible Comparisons test were applied. Mann–Whitney u test was applied to determine the significant difference in XlogP values.

## Supplementary Information

Below is the link to the electronic supplementary material.

Supplementary Material 1

## Data Availability

The data that support the findings of this study are available from the corresponding author upon reasonable request.

## Abbreviations

2D: Two-dimensional
3D: Three–dimensional-spheroids model
IC_50_: Half maximum inhibitory concentration
qRT-PCR: Quantitative real-time polymerase chain reaction
P53: Tumor suppressor protein
BAX: Encodes the pro-apoptotic protein
BCL2: Encodes the Bcl-2 protein
ELISA: Enzyme-linked immunosorbent assay
IL6: Interleukin-6
TNFα: A potent pro-inflammatory cytokine
MMP1: Matrix metallopeptidase 1
