# Supplementary material for: Assessment of cytotoxicity of some synthetic compounds against breast carcinoma spheroids with subsequent analysis of pro-apoptotic and gene expression
Source: Sci Rep. 2025 Nov 26;15:42543. doi: 10.1038/s41598-025-26942-w (PMC12663132; doi:10.1038/s41598-025-26942-w)
Supplement: Supplementary file 1 — Supplementary Material 1 [file 41598_2025_26942_MOESM1_ESM.docx]

**Supplementary data**

**Assessment of cytotoxicity of some synthetic compounds against breast carcinoma spheroids with subsequent analysis of pro-apoptotic and gene expression**

Khaled Mahmoud^1*^· Elham M. Youssef^2^·Farid M. Sroor^3^· Walid Fayad^1^

1. Drug Bioassay-Cell Culture Laboratory, Pharmacognosy Department, National Research Centre, Pharmaceutical and Drug Industries Division, Dokki, Giza, 12622, Egypt.
2. Biochemistry Department, National Research Centre, Giza, Egypt.
3. Organometallic and Organometalloid Chemistry Department, National Research Centre, Cairo 12622, Egypt

🖂 Khaled Mahmoud

[khaledmmh2003@yahoo.com](mailto:khaledmmh2003@yahoo.com)

**Table S1.** List of the 80 compounds.

| Plate location | | Chemical formula | | Chemical compounds name | | M.Wt | | |
| --- | --- | --- | --- | --- | --- | --- | --- | --- |
| A02 | | C17 H15 Cl N2 O3 S | | 3-(4-chloro-2,5-dimethoxyphenyl)-2-(methylthio)-3,4-dihydroquinazolin-4-one | | 362.836 | | |
| B02 | | C16 H14 N2 O2 S | | ethyl 4-(2-thioxo-2,3-dihydro-1H-benzimidazol-1-yl)benzoate | | 298.365 | | |
| C02 | | C16 H18 N2 S2 | | 2-({4-[(2-aminophenyl)thio]but-2-enyl}thio)aniline | | 302.464 | | |
| D02 | | C20 H13 N3 O2 | | 6-nitro-2,3-diphenylquinoxaline | | 327.342 | | |
| E02 | | C23 H28 N2 O10 | | methyl 5-ethoxy-2-{[5-ethoxy-3-(methoxycarbonyl)-4-(2-methoxy-2-oxoethyl)-2H-pyrrol-2-yliden]methyl}-4-(2-methoxy-2-oxoethyl)-1H-pyrrole-3-carboxylate | | 492.478 | | |
| F02 | | C14 H16 Cl N O S | | 2-{[(4-chlorophenyl)thio]methyl}quinuclidin-3-one | | 281.805 | | |
| G02 | | C20 H18 Cl N7 O3 S | | N-[4-(3-chlorophenyl)-2,5-dioxopiperazino]-2-{[4-methyl-5-(4-pyridinyl)-4H-1,2,4-triazol-3-yl]sulfanyl}acetamide | | 471.9272 | | |
| H02 | | C22 H28 Cl2 O2 | | 1,10-bis(4-chlorophenoxy)decane | | 395.367 | | |
| A03 | | C16 H12 Cl2 N4 O | | N4-(3,5-dichlorophenyl)-5-methyl-2-phenyl-2H-1,2,3-triazole-4-carboxamide | | 347.204 | | |
| B03 | | C9 H9 F3 N2 O | | N1-[2-(4-pyridyl)ethyl]-2,2,2-trifluoroacetamide | | 218.177 | | |
| C03 | | C16 H14 N4 O2 | | 1,2-di(3-methyl-2,3-dihydro-1,3-benzoxazol-2-yliden)hydrazine | | 294.313 | | |
| D03 | | C10 H6 Cl3 N3 O2 | | 2,2,2-trichloro-N-[3-(1,2,4-oxadiazol-3-yl)phenyl]acetamide | | 306.535 | | |
| E03 | | C21 H22 F2 N4 O2 S | | N2-[2-(tert-butylthio)ethyl]-4,6-di(4-fluorophenoxy)-1,3,5-triazin-2-amine | | 432.493 | | |
| F03 | | C15 H15 N3 O4 S3 | | ethyl 2-[(2,1,3-benzothiadiazol-4-ylsulfonyl)amino]-4,5-dimethylthiophene-3-carboxylate | | 397.499 | | |
| G03 | | C21 H14 F3 N O3 | | 3-[4-methoxy-2-(trifluoromethyl)-6-quinolyl]prop-2-ynyl benzoate | | 385.34 | | |
| H03 | | C19 H19 N3 O S | | N-(2,3-dihydro-1H-inden-2-yl)-2-[(5-methyl-1H-benzimidazol-2-yl)thio]acetamide | | 337.445 | | |
| A04 | | C15 H17 N3 O2 | | 1-(4-nitrophenyl)-4-(1H-pyrrol-1-yl)piperidine | | 271.318 | | |
| B04 | | C8 H10 N2 O3 | | 3-hydroxy-5-(hydroxymethyl)-2-methylisonicotinaldehyde oxime | | 182.178 | | |
| C04 | | C16 H21 N3 O S | | N1-[4-(tert-butyl)phenyl]-2-[(1-methyl-1H-imidazol-2-yl)thio]acetamide | | 303.428 | | |
| D04 | | C18 H18 N2 O2 | | 2-(1,3-benzodioxol-5-yl)-4,5,6,7-tetramethyl-1H-benzimidazole | | 294.3522 | | |
| E04 | | C12 H14 N2 O2 | | 2,3-diethoxyquinoxaline | | 218.255 | | |
| F04 | | C21 H19 N3 O4 | | 2-[6-(1,3-dioxo-2,3,3a,4,7,7a-hexahydro-1H-isoindol-2-yl)-2-pyridyl]-2,3,3a,4,7,7a-hexahydro-1H-isoindole-1,3-dione | | 377.398 | | |
| G04 | | C16 H14 N2 O4 S3 | | N-(2,3-dihydro-1,4-benzodioxin-6-yl)-5-(2-methyl-1,3-thiazol-4-yl)-2-thiophenesulfonamide | | 394.495 | | |
| H04 | | C18 H13 N O5 | | N3-(4H-1,3-benzodioxin-6-yl)-2-oxo-2H-chromene-3-carboxamide | | 323.303 | | |
| A05 | | C10 H11 N5 O3 S | | Ethyl 5-amino-1-[(4-methyl-1,2,3-thiadiazol-5-yl)carbonyl]-1H-pyrazole-4-carboxylate | | 281.295 | | |
| B05 | | C16 H11 F3 N2 O2 | | methyl 2-cyano-3-{1-[4-(trifluoromethyl)phenyl]-1H-pyrrol-2-yl}acrylate | | 320.269 | | |
| C05 | | C16 H16 N2 O . Cl H | | 1-benzyl-3-[2-(2-furyl)vinyl]-4,5-dihydro-1H-pyrazole hydrochloride | | 288.776 | | |
| D05 | | C14 H13 N O | | N-[1,1'-biphenyl]-4-ylacetamide | | 211.263 | | |
| E05 | | C12 H13 N O | | 2-[(dimethylamino)methylidene]indan-1-one | | 187.241 | | |
| F05 | | C11 H11 Cl N2 O2 S | | 3-[2-(4-chlorophenoxy)ethyl]-2-thioxoimidazolidin-4-one | | 270.739 | | |
| G05 | | C17 H15 N3 O4 S3 | | methyl 3-{[4-(2,3-dihydroimidazo[2,1-b][1,3]thiazol-6-yl)anilino]sulfonyl-2-thiophenecarboxylate | | 421.521 | | |
| H05 | | C3 H7 N O2 S2 | | 2-(methylsulfonyl)ethanethioamide | | 153.22 | | |
| A06 | | C12 H10 N4 O4 | | 3,3'-dinitro[1,1'-biphenyl]-4,4'-diamine | | 274.235 | | |
| B06 | | C11H11N3OS | | 1-(5-anilino-1,2,4-thiadiazol-3-yl)acetone | | 233.29 | | |
| C06 | | C14 H15 NO4 | | N-(2-furylmethyl)-3,4-dimethoxybenzamide | | 261.276 | | |
| D06 | | C9 H8 Cl N O6 | | 3-chloro-2,6-dimethoxy-5-nitrobenzoic acid | | 261.6162 | | |
| E06 | | C20 H14 N4 | | 2,3-diphenyl-2H-pyrido[1,2-b][1,2,4]triazine-2-carbonitrile | | 310.359 | | |
| F06 | | C17 H17 Cl2 N3 O3 S | | N1-(2,5-dichlorophenyl)-2-[3-(4-methoxyphenoxy)propanoyl]hydrazine-1-carbothioamide | | 414.311 | | |
| G06 | | C15 H12 F N3 O S | | N'-(2H-1,4-benzothiazin-3-yl)-4-fluorobenzohydrazide | | 301.344 | | |
| H06 | | C11 H8 O2 S | | 4-(3-thienyl)benzoic acid | | 204.2482 | | |
| A07 | | C19 H16 Cl N O2 S2 | | 2-(2,6-dimethylanilino)-2-oxoethyl 3-chlorobenzo[b] thiophene-2-carbothioate | | 389.925 | | |
| B07 | | C9 H10 Cl N O4 S2 | | 3-chloro-2-({[(1methylethylidene)amino]oxy}carbonyl)-4-(methylsulfonyl)thiophene | | 295.766 | | |
| C07 | | C11 H13 N3 O2 S | | 4-(3,5-dimethyl-1H-pyrazol-1-yl)benzene-1-sulfonamide | | 251.309 | | |
| D07 | | C20 H17 Cl2 N5 O3 S | | 4-(4-chloro-2,5-dimethoxyphenyl)-5-{2-[3-(4-chlorophenyl)-1,2,4-oxadiazol-5-yl]ethyl}-4H-1,2,4-triazole-3-thiol | | 478.358 | | |
| E07 | | C11 H11 N3 O3 S2 | | 4-[2-(2-thienylcarbonyl)hydrazino]benzenesulfonamide | | 297.358 | | |
| F07 | | C20 H18 Cl2 N2 O4 S | | N4-{2-[(4-methylphenyl)sulfonyl]ethyl}-3-(2,6-dichlorophenyl)-5-methylisoxazole-4-carboxamide | | 453.344 | | |
| G07 | | C18 H15 N3 O4 S | | N-[3-(aminosulfonyl)phenyl]-2-phenoxynicotinamide | | 369.4 | | |
| H07 | | C9 H7 Br2 Cl2 N O | | N1-(2,6-dibromo-4-methylphenyl)-2,2dichloroacetamide | | 375.874 | | |
| A08 | | C16 H16 N4 O4 | | 3-[(2-morpholinoanilino)carbonyl]-2-pyrazinecarboxylic acid | | 328.326 | | |
| B08 | | C14 H16 N2 O2 S | | N-hexahydro[1,3]thiazolo[3,4-a]pyridin-3-ylidene-1,3-benzodioxol-5-amine | | 276.358 | | |
| C08 | | C13 H9 N O6 | | 2-acetylphenyl 5-nitro-2-furoate | | | 275.215 | |
| D08 | | C17 H16 Cl N3 O | | N-(4-chlorophenyl)-N'-[2-(1H-indol-3-yl)ethyl]urea | | | 313.786 | |
| E08 | | C13 H10 F3 N5 O3 | | N'-({[4-(trifluoromethoxy)anilino]carbonyl}oxy) pyrazine-2-carboximidamide | | | 341.248 | |
| F08 | | C18 H21 F3 N4 O S | | 1-(phenylthio)-3-{4-[4-(trifluoromethyl)pyrimidin-2-yl]piperazino}propan-2-ol | | | 398.451 | |
| G08 | | C16 H12 F3 N3 O2 | | N-[4-(cyanomethyl)phenyl]-N'-[4-(trifluoromethoxy) phenyl]urea | | | 335.284 | |
| H08 | | C13 H16 Br N O3 | | 5-[(4-bromophenoxy)methyl]-3-isopropyl-1,3-oxazolan-2-one | | | 314.177 | |
| A09 | | C18 H15 N3 O3 | | N2-(3-phenyl-1H-pyrazol-5-yl)-2,3-dihydro-1,4-benzodioxine-2-carboxamide | | | 321.335 | |
| B09 | | C10 H7 N3 O3 | | 5-(4-pyridylmethylidene)hexahydropyrimidine-2,4,6-trione | | | 217.183 | |
| C09 | | C13 H11 N5 O4 | | 3-({[6-(acetylamino)-3-pyridinyl]amino}carbonyl)-2-pyrazinecarboxylic acid | | | 301.261 | |
| D09 | | C16 H23 N3 O5 | | 1-[2-hydroxy-3-(4-methyl-2-nitrophenoxy)propyl]piperidine-4-carboxamide | | | 337.374 | |
| E09 | | C19 H22 Cl N3 O2 | | N-(5-chloro-2-methoxyphenyl)-N'-(4-piperidinophenyl) urea | | | 359.855 | |
| F09 | | C12 H7 F3 N4 O S | | 2-(4-methyl-1,2,3-thiadiazol-5-yl)-5-[2-(trifluoromethyl) phenyl]-1,3,4-oxadiazole | | | 312.274 | |
| G09 | | C19 H25 N3 O4 | | 1-{[(1,1,3,3,6-pentamethyl-7-nitro-2,3-dihydro-1H-inden-5-yl)amino]methyl}pyrrolidine-2,5-dione | | | 359.423 | |
| H09 | | C14 H12 N2 O4 | | methyl 5-(2,2-dicyanovinyl)-2,4-dimethoxybenzoate | | | 272.259 | |
| A10 | | C18 H16 Cl F N2 O4 S2 | | N4-[4-(propylsulfonyl)-3-thienyl]-3-(2-chloro-6-fluorophenyl)-5-methylisoxazole-4-carboxamide | | | 442.917 | |
| B10 | | C8 H10 N2 O | | 2-phenylacetohydrazide | | | 150.18 | |
| C10 | | C15 H9 Cl2 F3 O S | | S-[3-(trifluoromethyl)benzyl] 3,4-dichlorobenzene carbothioate | | | 365.201 | |
| D10 | | C18 H15 Cl F N3 O3 | | 3-(2-chloro-6-fluorophenyl)-N'-(4-methoxyphenyl)-5-methyl-4-isoxazolecarbohydrazide | | | 375.785 | |
| E10 | | C20 H17 F3 N2 O3 | | O6-[3-(trifluoromethyl)benzoyl]-2,2-dimethyl-2H-chromene-6-carbohydroximamide | | | 390.359 | |
| F10 | | C27 H16 F3 N3 O5 | | 4-(1,3-dioxo-2,3-dihydro-1H-inden-2-yliden)-6-(2-pyridyl)-2-[4-(trifluoromethoxy)phenyl]perhydropyrrolo [3,4-c]pyrrole-1,3-dione | | | 519.433 | |
| G10 | | C12 H8 F3 N O3 | | 5-[2-(trifluoromethoxy)phenyl]-2-furaldehyde oxime | | | 271.193 | |
| H10 | | C14 H17 N5 O6 | | ethyl 4-[2-(2,4-dinitrophenyl)hydrazono]piperidine-1-carboxylate | | | 351.317 | |
| A11 | | C27 H17 Cl N4 S2 | | 8-[(4-chlorobenzyl)sulfanyl]-3,4-diphenylpyrimido [4',5':4,5]thieno[2,3-c]pyridazine | | | 497.044 | |
| B11 | | C11 H5 F6 N3 O2 | | 1-[2-nitro-4-(trifluoromethyl)phenyl]-3-(trifluoromethyl)-1H- pyrazole | | | 325.1675 | |
| C11 | | C15 H10 Cl F3 N2 O3 | | N'1-(4-chlorobenzoyl)-4-(trifluoromethoxy)benzene-1-carbohydrazide | | | 358.702 | |
| D11 | | C22 H21 F3 N2 O6 S | | N'1-{2-[(2-oxo-4-propyl-2H-chromen-7-yl) oxy] propanoyl}-3-(trifluoromethyl)benzene-1-sulfonohydrazide | | | 498.476 | |
| E11 | | C18 H17 Cl N2 O2 | | 2-amino-4-(4-chlorophenyl)-7,7-dimethyl-5-oxo-5,6,7,8-tetrahydro-4H-chromene-3-carbonitrile | | | 328.797 | |
| F11 | | C13 H13 N3 O3 S | | 3-[(anilinocarbonyl)amino]benzenesulfonamide | | | 291.33 | |
| G11 | | C12 H11 N3 O2 | | 3-[4-(acetylamino)phenyl]-2-cyanoacrylamide | | | 229.238 | |
| H11 | | C10 H8 F3 N3 O | | N2-(2-furylmethyl)-4-(trifluoromethyl)pyrimidin-2-amine | | | 243.187 | |

**XlogP values for the eighty compounds and subsequent % inhibition values for the 2D and 3D**

| Compounds | XLogP | % inhibition | % inhibition |
| --- | --- | --- | --- |
|  |  | 2D | 3D |
| A02 | 4.33 | 94.3 | 67.1 |
| B02 | 3.99 | 100 | 35.7 |
| C02 | 3.61 | 100 | 87.3 |
| D02 | 5.05 | 5.2 | -8 |
| E02 | 0.11 | 83.2 | -10.8 |
| F02 | 2.5 | 100 | 52 |
| G02 | 0.66 | 0 | -9.9 |
| H02 | 7.75 | 68.7 | 3.5 |
| A03 | 4.7 | 94.2 | 39.1 |
| B03 | 1.1 | 0 | 25.5 |
| C03 | 5.36 | 86.6 | 3.4 |
| D03 | 3.12 | 11.1 | 23.5 |
| E03 | 6.1 | 80.3 | 41.8 |
| F03 | 4.53 | 70 | 91.5 |
| G03 | 5.16 | 49.8 | 21.3 |
| H03 | 3.54 | 88.5 | 15.8 |
| A04 | ---- | 2.5 | 43.1 |
| B04 | 0.57 | -58.5 | 49.3 |
| C04 | 3.21 | 14.5 | 28.6 |
| D04 | 4.59 | 100 | 67.9 |
| E04 | 3.06 | 62.6 | 42 |
| F04 | 2.09 | 46.9 | 29.8 |
| G04 | 3.62 | 0 | 6.3 |
| H04 | 2.19 | 46 | 28 |
| A05 | ---- | 62.3 | 49 |
| B05 | 3.46 | 91.3 | 42.1 |
| C05 | ---- | 62.9 | 48.6 |
| D05 | 2.62 | 49.7 | 50.9 |
| E05 | 1.67 | 47.5 | 41.4 |
| F05 | 1.91 | 96.9 | 31.6 |
| G05 | 3.05 | 65.7 | 35.1 |
| H05 | -1.8 | 24.1 | 19.3 |
| A06 | 1.59 | 82.4 | 60.8 |
| B06 | 3.81 | 14.8 | 20.4 |
| C06 | 1.27 | 0 | 35.8 |
| D06 | 2.06 | 10.4 | 40.8 |
| E06 | 4 | 26.7 | 55 |
| F06 | 3.92 | 80.1 | 88 |
| G06 | 3.92 | 93.4 | 52.3 |
| H06 | 3.19 | 7.8 | 26.1 |
| A07 | 5.32 | 36.5 | 75.1 |
| B07 | ---- | 0 | 34.9 |
| C07 | 1.82 | 0 | 55.4 |
| D07 | 6.5 | 88.7 | 50.1 |
| E07 | 1.08 | 0 | 36.2 |
| F07 | 4.06 | 4.4 | 54.2 |
| G07 | 2.5 | 0 | 35.1 |
| H07 | 4.26 | 65.3 | 40.6 |
| A08 | 0.45 | 17.2 | 49.3 |
| B08 | 3.48 | 8.5 | 53.1 |
| C08 | ---- | 66.3 | 30.8 |
| D08 | 2.96 | 97.7 | 74 |
| E08 | 1.64 | 84.1 | 40.2 |
| F08 | 3.69 | 100 | 61.6 |
| G08 | 4.02 | 82.8 | 56.2 |
| H08 | 2.99 | 18 | 49 |
| A09 | 2.59 | 100 | 52.2 |
| B09 | -0.8 | 12.3 | 59.2 |
| C09 | -1.1 | 9.9 | -23 |
| D09 | 0.16 | 7.6 | 50 |
| E09 | 3.96 | 28.3 | 50.7 |
| F09 | ---- | 9.9 | 14.2 |
| G09 | ---- | 100 | 66.5 |
| H09 | 1.87 | 86.2 | 80.8 |
| A10 | 3.79 | 53.8 | 68.6 |
| B10 | 0.64 | 0 | 48.2 |
| C10 | 6.19 | 100 | 33.8 |
| D10 | 3.55 | 52.1 | 51.7 |
| E10 | 5.16 | 100 | 67.5 |
| F10 | 2.58 | 100 | 60.3 |
| G10 | 4.04 | 100 | 54.4 |
| H10 | 2.13 | 27.5 | 60.6 |
| A11 | 8.79 | 44.2 | 46.4 |
| B11 | ---- | 0 | 30.5 |
| C11 | 4.39 | 83.1 | 66.5 |
| D11 | 3.56 | 35.5 | 40.6 |
| E11 | 2.06 | 93.7 | 66.4 |
| F11 | 1.26 | 0 | 60.4 |
| G11 | 0.09 | 15.6 | 60.8 |
| H11 | 1.8 | 0 | 68.5 |
